# Multicenter, Prospective, Randomized, Single Blind, Cross-Over Study on the Effect of a Fixed Combination of Citicoline 500 mg Plus Homotaurine 50 mg on Pattern Electroretinogram (PERG) in Patients With Open Angle Glaucoma on Well Controlled Intraocular Pressure

**DOI:** 10.3389/fmed.2022.882335

**Published:** 2022-04-29

**Authors:** Gemma Caterina Maria Rossi, Teresa Rolle, Annalisa De Silvestri, Dario Sisto, Alberto Mavilio, Alessandra Venera Mirabile, Alessia Paviglianiti, Beatrice Strano, Erica Picasso, Gian Maria Pasinetti, Giovanni Milano, Giulio Ruberto

**Affiliations:** ^1^Department of Surgical Sciences, University Eye Clinic, Fondazione IRCCS Policlinico S. Matteo, Pavia, Italy; ^2^Department of Surgical Sciences, School of Medicine, University of Turin, Turin, Italy; ^3^Clinical Epidemiology and Biometric Unit, Scientific Direction, Fondazione IRCCS Policlinico S. Matteo, Pavia, Italy; ^4^Department of Neuroscience, Eye Clinic, Aldo Moro University of Bari, Bari, Italy; ^5^ASL of Brindisi, Brindisi, Italy; ^6^Eye Unit, Istituto Beato Palazzolo, Bergamo, Italy

**Keywords:** glaucoma, citicoline, homotaurine, pattern electroretinogram (PERG), neuroprotection, quality of life, visual field, Retimax

## Abstract

**Purpose:**

To evaluate the potential beneficial and synergistic effects of oral intake of a fixed combination of citicoline 500 mg plus homotaurine 50 mg (CIT/HOMO) on retinal ganglion cell (RGC) function in subjects with glaucoma using pattern electroretinogram (PERG) and to investigate the effects on visual field and quality of life.

**Methods:**

Consecutive patients with primary open-angle glaucoma with controlled IOP (<18 mmHg) receiving beta-blockers and prostaglandin analogs alone or as combination therapy (fixed or un-fixed); with stable disease (progression no more than −1 dB/year at the visual field MD); and an early to moderate visual field defect (MD < −12 dB) were randomized to: arm A. topical therapy + CIT/HOMO for 4 months, 2 months of wash out, 4 months of topical therapy alone; arm B. topical therapy alone for 4 months, topical therapy + CIT/HOMO for 4 months, 2 months of wash out. All patients underwent 4 visits: complete ocular examination, visual field, PERG and quality of life assessment (NEI-VFQ25) were performed at each visit.

**Results:**

Fifty-seven patients completed the study: 26 in group A and 31 in group B. At the end of the intake period, PERG's P50 and N95 waves recorded a greater amplitude. The increase was statistically significant in the inferior and superior P50 waves amplitude: 0.47 μV (95%CI, 0.02–0.93; *p* = 0.04) and 0.65 μV (95% CI, 0.16–1.13; *p* = 0.009), respectively, and in the inferior N95 wave amplitude 0.63 μV (95% CI, 0.22–1.04; *p* = 0.002). A significantly shorter peak time of 3.3 μV (95% CI, −6.01– −0.54; *p* = 0.01) was observed for the superior P50 wave only.

**Conclusions:**

Daily oral intake of the fixed combination CIT/HOMO for 4 months improved the function of inner retinal cells recorded by PERG in the inferior and in the superior quadrants, independently from IOP reduction. This interesting association could represent a valid option for practicing neuromodulation in patients with glaucoma to prevent disease progression.

## Introduction

The term “glaucoma” includes a group of diseases characterized by progressive optic nerve degeneration that results in irreversible blindness. Glaucoma is a progressive and multifactorial neurodegenerative disease involving retinal ganglion cells (RGCs) with their axons and can be considered a neurodegenerative disorder involving both the eye and the brain. The eye, infact, is an extension of the central nervous system (CNS) and its degenerative diseases lead to progressive loss of vision recognizing, for glaucoma, causes other than intraocular pressure (IOP) such as genetic predisposition, environmental factors, metabolic alterations and inflammatory processes (1).

This explains the need for new diagnostic methods and biomarkers and the use of alternative options to treat glaucoma. Research focuses primarily on the role of inflammation in the development of glaucoma and, consequently, on neuroprotective and neuromodulatory treatments to prevent or avoid disease progression.

Currently, the main strategy to treat glaucoma is the lowering of IOP, since this treatment has proven effective in preserving visual function in both the early and late stages (2–5). However, it is not always effective and sufficient to avoid the progression of glaucoma: for this reason, experimental models of glaucoma have been studied and have shown that RGCs die from apoptosis and that their fate is determined by factors, other than IOP, depending on a cross-dialogue between proapoptotic signals and survival promoters (6).

Based on this experimental data, several compounds have been studied and have shown to be neuroprotective in experimental animal models of glaucoma, but there is a lack of clinical studies on humans to confirm *in vitro* effects (7).

Citicoline has shown a neuromodulator effect in patients with “controlled IOP” glaucoma treated with citicoline oral solution (500 mg/day, daily for 4 months divided by 2-month non-treatment periods) who achieved a reduction in the rate of progression of the visual field defect (8): this effect has been explained to the enhancement of the RGCs function and neural conduction along the postretinal visual pathways due to citicoline assumption. Other previous studies have shown that citicoline acts as a neuroenhancer in both the central nervous system and the eye (9–13).

Regarding homotaurine, it acts against oxidative damage to DNA, exercising antifibrillogen activity, antinociceptive and analgesic activity, and resulting as a neuprotector and neutrotropic molecule; moreover it is able to inhibit the formation of the β-amyloid plaque (14) responsible for neuronal cells apoptosis and for numerous neurodegenerations in the CNS, but also in the ophthalmic field (15).

Given that the degeneration of the neurons of the RGCs occurs due to various factors (16), it is probable that by combining several neuromodulatory molecules, which act on the various causes of cell death, it is possible to obtain a synergistic action and an increase in their neuroprotective efficacy. For these reasons, studies are underway to evaluate the potential of associations of molecules with different mechanisms of action and verify their real synergistic effects in neuromodulation.

Some *in vitro* studies on mixed retinal cells from Wistar rats have shown a synergistic action of citicoline and homotaurine in reducing cytotoxicity and apoptosis (17) in an *in vitro* model of neurotoxicity, but studies on humans have yet to be done.

To date, in Italy, Homotaurine and Citicoline are substances allowed as food supplements (Ministry of Health “Other nutrients and other substances with nutritional or physiological effects”, Revision February 2017) and a fixed combination of these two molecules is available in tablets.

The main objective of the present study was to evaluate the potential beneficial effects of supplementing a fixed combination of Citicoline 500 mg plus Homotaurine 50 mg on RGCs function in subjects with glaucoma treated with topical medical therapy alone by electroretinogram; and evaluate any effects on visual field examination and vision-related quality of life.

## Materials and Methods

**Figure 1:**
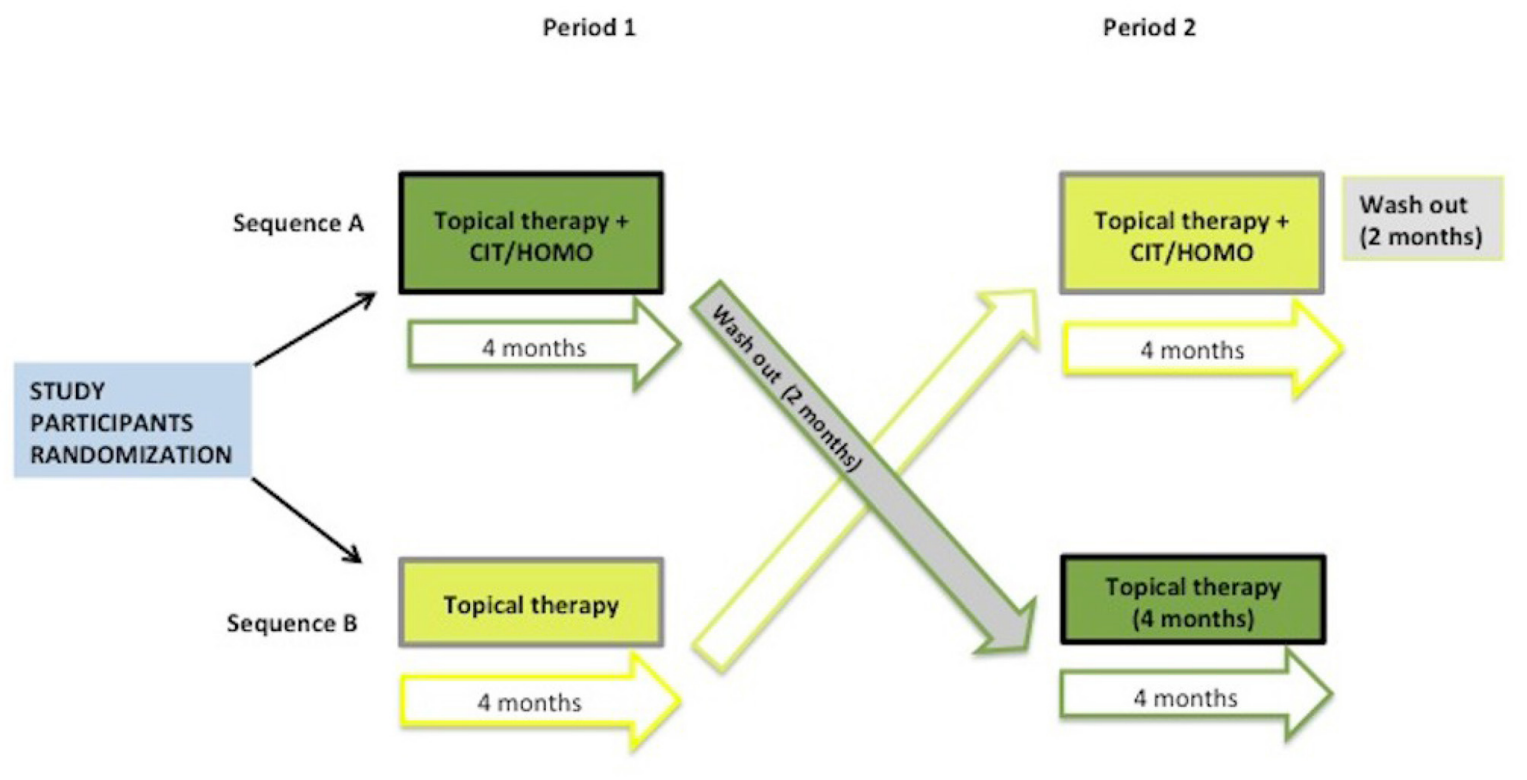
Study design.

A multicentre, randomized, 2-sequence, 2-period, 2-treatment crossover study (Figure 1) with blind outcome assessor was conducted. A cross-over study was designed to limit intraindividual variability. Blinding was not considered because the patient cannot interfere with the measurement of the pattern-electroretinogram (PERG).

**Figure 2:**
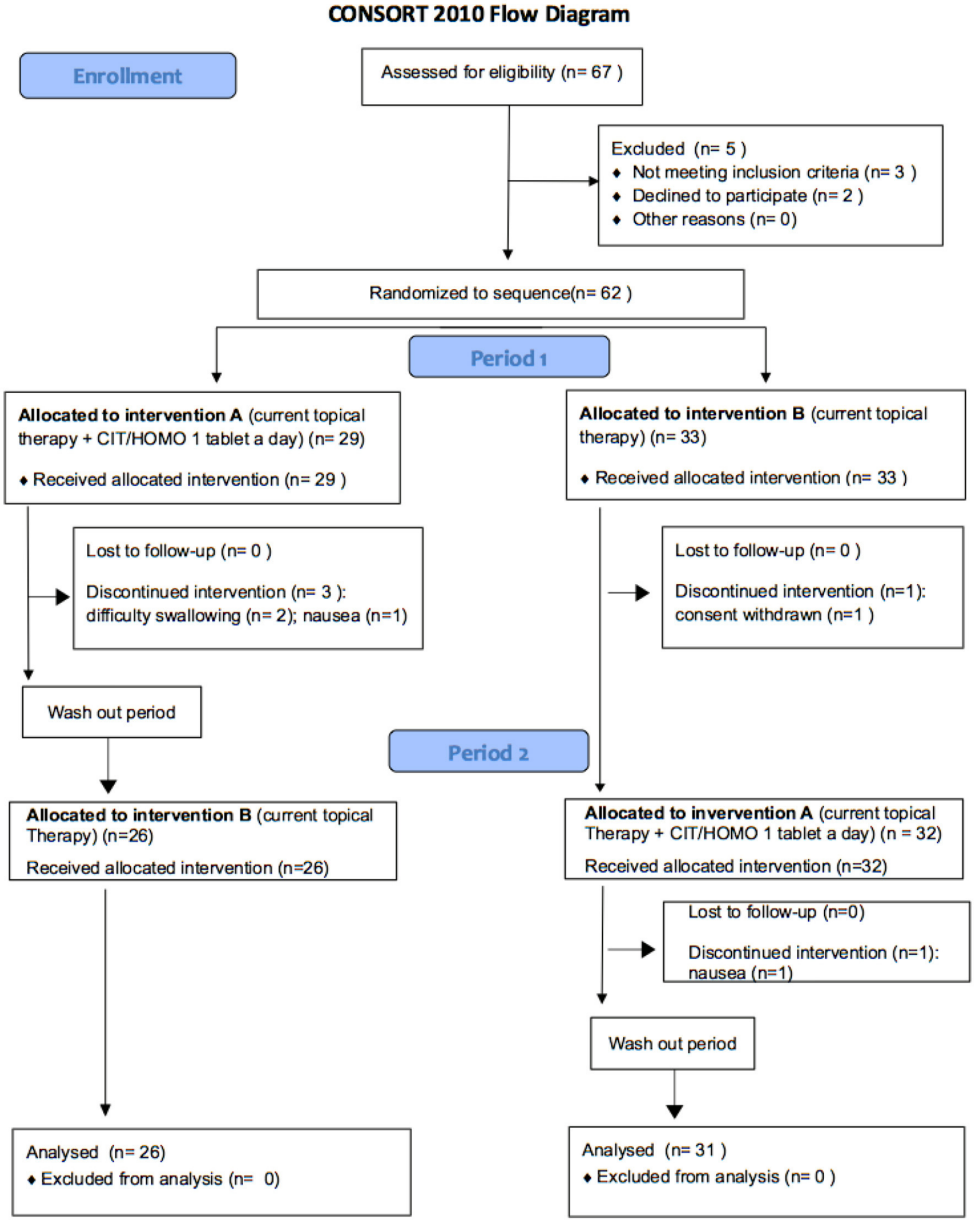
CONSORT flow diagram.

The study was conducted according to the recommendations of the Declaration of Helsinki (revision 2000, Edinburg) and of the Italian legislation of Good Clinical Practice (Ministerial Decree of 15 July 1997 and subsequent amendments) subject to the approval of the Ethics Committee of the IRCCS Policlinico San Matteo Foundation of Pavia (prot. 2015000565); it complied with CONSORT 2010 guidelines (Figure 2) and it was registered on ClinicalTrials.gov (NCT identifier 04422743).

Patients were enrolled from the “glaucoma center” of four Italian Eye Clinics: Pavia, Turin, Bari and Brindisi.

All eligible patients were asked to complete the study and sign the informed consent form: signed informed consent was obtained from all individual participants included in the study. The study was not supported by any industry nor received any other form of financial grant or sponsorship. At the request of the Ethics Committee, FB vision (the Italian company that produces the CIT/HOMO fixed association) provided free all product packages, for all patients in the study, for the entire duration of the study. Patients who no longer wished to participate after signing informed consent or any other condition that, in the investigator's clinical judgment, made further study participation unacceptable for that individual patient were withdrawal criteria.

### Eligibility Criteria

**Figure 3:**
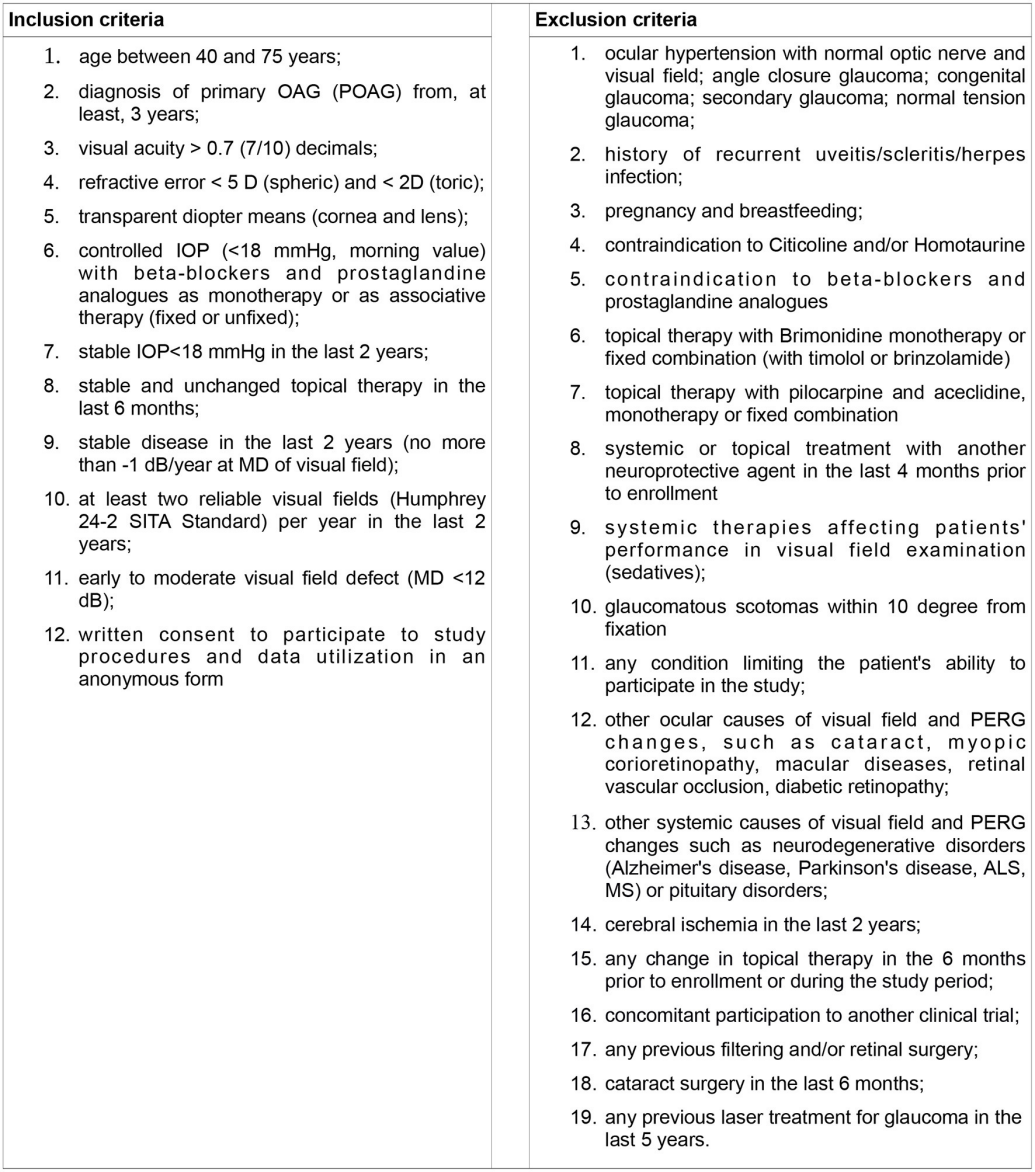
Inclusion and exclusion criteria.

Inclusion and exclusion criteria are shown in Figure 3.

### Objectives

#### Primary Objective

To compare the effects of adding to standard topical therapy the fixed combination of Citicoline 500 mg plus Homotaurine 50 mg (CIT/HOMO; Neuprozin®), one tablet per day, on PERG after four months of therapy, compared to topical therapy alone (standard of care = SOC).

#### Secondary Objectives

To compare the two treatments (CIT/HOMO+ SOC vs. SOC alone) in terms of:

- visual acuity over time
- visual field changes over time
- quality of life perception (NEI VFQ25 questionnaire) over time
- safety (incidence of adverse events)

### Study Treatments

#### Standard Treatment, Currently Available in the Clinical Practice

The treatment of glaucoma is based on the reduction of IOP, the standard treatment of glaucoma consists in the administration of eye drops that reduce the intraocular pressure, in particular by prescribing prostaglandine derivatives and beta-blockers which are the most effective agents (1). As defined in the Eligibility Criteria (Figure 3), selected patients were required to be on topical therapy with beta-blockers, twice daily (carteolol 2% or timolol 0.5%) and/or prostaglandin derivatives, once daily (bimatoprost, latanoprost, travoprost or tafluprost) for at least 1 year.

#### Investigational Treatment

Patients were randomized (1:1) into two arms (= treatment sequences) (Figure 1):

- Arm A: CIT/HOMO + topical therapy followed by no supplement, other than the standard treatment
- Arm B: no supplementation followed by CIT/HOMO in addition to the standard topical treatment

The dosage of CIT/HOMO was one tablet per day, in the morning, for 4 months.

Ultrapure Citicoline is a molecule normally contained in our body and plays a fundamental role in the processes of formation and repair of the membranes of nerve cells and in the propagation of nerve impulses with a facilitating effect on the cerebral energy metabolism (12, 13, 16).

Homotaurine is an amino acid extract derived from algae that can reach the central nervous system and contribute to the propagation of the nerve impulse (14, 15).

### Detailed Study Procedures

#### Study Design and Scheduled Visit

Four visits (V0-V3) were planned for each subject on a 10 months period, as resumed in Figure 1.

V0 = randomization
V1 = Arm A: end of SOC+ CIT/HOMO (4 months)
Arm B: end of SOC (4 months)
V2 = Arm A: end of wash out (2 months)
Arm B: end of SOC+ CIT/HOMO (4 months)
V3 = Arm A: end of SOC alone (4 months)
Arm B: end of wash out (2 months)

The statistician (DSA) generated the random allocation sequence. One resident (PE) delivered CIT/HOMO to patients based on randomization.

#### Screening Procedures

The inclusion/exclusion criteria were verified after careful examination of the patients' medical records. If suitable, at the first clinical visit following the start of the study, the patient was informed of the protocol and his/her written consent was required both for participation in the study and for the use of his /her clinical data for scientific evaluations.

#### Planned Assessments

At all visit (V0–V3) all patients underwent the following routine procedures:

- complete eye examination
  ° Visual acuity (VA) (decimals)
  ° Anterior segment evaluation (anterior chamber, lens and angle)
  ° IOP (Goldmann tonometry)
  ° evaluation of the optic nerve with indirect lens (Volk 90)
- pattern electroretinogram (PERG)
- visual field examination (Humphrey 24-2 sita- standard)
- quality of life assessment by NEI VFQ 25 questionnaire.

#### Other Variables

Other variables collected for each subject at baseline/over time were: date of birth (month/year) and gender, therapy (systemic and topical), medical history (systemic and ocular, i.e. previous laser/surgery).

#### Single Blind Procedures

All personnel involved in performing the ophthalmic examination, the visual field examination, optic nerve evaluation, IOP measurement, and PERG test were blind to the patient's treatment period; the data analyst was also blind to the treatment group.

### Adverse Events

Adverse events (AEs), both systemic and topical, were collected at each visit, and patients were asked about the comfort of therapy and any side effects.

For each AEs the following information were recorded in the patient's medical chart: nature of adverse event: date and time of occurrence and disappearance (i.e., duration); intensity: mild, moderate or severe; frequency: once, continuous or intermittent; decision regarding study: continuation or withdrawal; relation to the study medication; measures undertaken to treat it.

AEs were treated according to the usual clinical practice. In the event of a serious adverse event the drug was discontinued.

### Study Definitions and Diagnostic Criteria

#### Glaucoma Diagnosis

Diagnosis of glaucoma required: IOP > 21 mmHg on at least two consecutive visits at the time of first diagnosis, presence of a glaucomatous optic nerve head (ONH) confirmed by expert examination, and at least three reliable full-threshold Humphrey 24-2 visual field performed on different days showing a glaucomatous defect or a suspected glaucomatous defect.

The diagnosis was made (or confirmed, if the patient is referred from another center) by a senior ophthalmologist who worked at the Eye Clinic of each Center.

#### Ocular Examination

Visual acuity was determined in decimal units (Snellen).

The anterior and posterior segments, with particular attention to the retina and the appearance of the optic nerve, were performed with the slit lamp.

The IOP was measured with Goldmann tonometer. The ophthalmologist who measured the IOP was blinded to the treatment period.

#### Visual Field Examination

The Humphrey Visual Field is a special automated procedure used to perform perimetry; the test measures the entire area of peripheral vision that can be seen while the eye is focused on a central point. During this test, lights of varying intensities appear in different parts of the visual field while the patient's eye is focused on a certain spot. The perception of these lights is charted and then compared to results of a healthy eye at the same age of the patient in order to determine if any damage has occurred. This procedure is performed quickly and easily in about 10 min, and is effective in diagnosing and monitoring the progress of glaucoma. Main parameters to evaluate damage and progression or not are: mean defect and pattern standard deviation (MD and PSD, decibels), Glaucoma hemifield test (GHT, qualitative description of the examination defined normal, borderline, outside normal limits), and the visual field index (VFI, a global index that assigns a number between 1 and 100% based on an aggregate percentage of visual function with 100% being a perfect age-adjusted visual field).

All patients were submitted to 24-2 SITA-Standard Humphrey Visual Field examination.

#### Pattern Electroretinogram (PERG)

PERG is an objective and direct measure of inner retinal function correlated with RGCs activity, it has been shown that PERG is able to document and predict early glaucomatous changes (18, 19).

The PERG is a retinal biopotential evoked when a temporally modulated patterned stimulus (checkerboard or grating) of constant mean luminance is viewed.

PERG is a very small signal, typically in the region of 0.5–8 μV depending on the characteristics of the stimulus, and PERG recording is technically more demanding than conventional ERG.

PERG was recorded according to the International Society for Clinical Electrophysiology of Vision guidelines, using Retimax®, glaucoma Hemifield PERG test (CSO, Florence, Italy), a device that has a large 55' screen with an hardware which eliminates the luminance influence on PERG and that repeats the stimulation twice in each session, using a specific software (hemifield stimulation) that allows the intra-individual variability assessment. The test records the wave in the center, in the superior and in the inferior hemifield.

##### Transient PERG

At low temporal frequencies (six reversals per second or less, equivalent to 3 Hz or less i.e. 1–2 Hz) a transient PERG is obtained.

**Figure 4:**
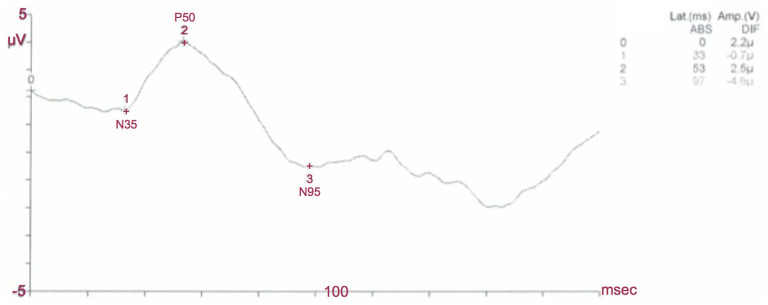
Normal PERG. In this normal subject the P50 latency is 53 ms, the P50 amplitude is 2,5 μV, the N95 latency is 97 ms and the N95 amplitude is 4,6 μV: PERG amplitudes and latencies of P50 and N95 waves are within normal limits.

The waveform is characterized by a small initial negative (N) component, at approximately 35 ms, which will be referred to as N35. This is followed by a later and larger positive (P) component (45–60 ms) which is typically denoted as P50. This positive portion of the waveform is followed by a large negative component at about 90–100 ms (N95) (Figure 4).

For the transient PERG, amplitude measurements are made between peaks and troughs: The P50 amplitude is measured from the trough of N35 to the peak of P50; the N95 amplitude is measured from the peak of P50 to the trough of N95. Latency measurements should be taken from the onset of the stimulus to the peak of the component concerned. The highest absolute amplitude point on a waveform will not always be appropriate for the definition of the peak if there is contamination from muscle activity or other artifacts.

##### Steady-State PERG

At higher temporal frequencies [15 rev/s (7.5 Hz)], a “steady-state” PERG is evoked.

A horizontal bars stimulus with a spatial frequency of 1.6 cycles/degree is presented on a plasma display that underlies the 30 central degrees at a distance of 100 cm.

The waveform becomes roughly sinusoidal, and Fourier analysis is required to determine the 15 Hz peak, the second harmonic, that is, the harmonic that has a frequency twice that of the stimulus.

The second harmonic contains the information of the bioelectric signal generated largely by the retinal ganglion cells, namely the amplitude (expressed in μV) and the phase (in pico-rad), whose values are automatically provided by the instrument.

##### Clinical Protocol: Patient Preparation

Pupils: PERG was recorded without dilating pupils to preserve accommodation.

Fixation: A central fixation point was located at the center of the screen

Refraction: Because of the nature of the stimulus, PERG examination was performed with optimal visual acuity at the testing distance. Patients wear the appropriate optical correction for the test distance.

Recording: Proportional distribution was used until a stable waveform was obtained. It included a minimum of 150 responses (more would be needed in a “noisy” subject). At least two trials for each stimulus condition should be obtained to confirm reproducibility.

The system complies with the European directive 93/42 technical standard IEC 601-1 CEI 62-5 quality system UNI EN ISO 9001

The DTL electrodes for PERG (Bionen S.a.s. Firenze) were used to register the examination.

#### NEI VFQ 25 Item

The patients' quality of life was examined with the Italian version of the 25 item National Eye Institute Visual Function Questionnaire (20). The 25 item NEI-VFQ is a vision-targeted non-disease-specific instrument designed to measure the impact of some ocular disorders on vision related quality of life. Depending on the item, responses to this questionnaire pertain to the frequency or severity of a symptom or a problem with the functioning. The NEI-VFQ scores can range from 0 to 100 with lower scores indicating more problems or symptoms.

### Statistical Issues

#### Primary Endpoint

Amplitude of P50 wave at 100 cm distance, at the transient PERG, as measured at the end of each study period.

#### Elements for Sample Size Calculations or Study Power

A two-sided t-test achieves 81% power to infer that the mean difference is not 0 (zero) when the total sample size of a 2x2 cross-over design is 66, the actual mean difference is 0,5, the square root of the within mean square error is 1,0, and the significance level is 0,05. (Estimates from: Parisi et al. Evidence of the neuroprotective role of citicoline in glaucoma patients. Prog Brain Res. 2008;173:541-54. 10.1016/S0079-6123(08)01137-0.)

Considering the potential for losses to follow-up, we plan to enroll 72 patients (36 per arm=sequence). Due to COVID-19 pandemy, the enrollment was interrupted at 62 patients.

#### Analysis Plan

Sequence influence and period influence was preliminary tested by a mixed model with random effects per eye, patient and period, and fixed effect for interaction between treatment and sequence; if the interaction was not significant (p <0.05), this indicate no carry-over effect and the primary endpoint was compared by means of t-test.

Full statistical analysis plans (SAPs) was developed prior to data analysis, as for GCP. Briefly, descriptive statistics were obtained for all variables assessed in the study population. Mean and standard deviation were used for normally distributed variables, mean and interquartile range for skewed distributions, proportions for categorical variables. Groups were compared by means of parametric or nonparametric tests for quantitative variables and Pearson's χ2 test (Fisher exact test where appropriate) for categorical variables. In all cases, two-tailed tests were applied. *P* < 0.05 was considered significant.

Multilevel generalized linear models were used to assess the effect of treatment, sequence and period of:

• PERG variables other-than-primary-endpoint
• visual acuity
• visual field changes over time (yes vs no)
• NEI VFQ25 total score and individual subscales

We also fitted models including age, visual field, IOP and type of therapy (combined or monotherapy) and their interaction with NP to assess whether the effect of NP varied based on these parameters. These models are also multilevel (eyes and patients) to take into account the clustered nature of the data.

## Results

Sixty-two patients were enrolled and signed informed consent, but five patients left the clinical trial: three in arm A (two for difficulty swallowing, one for nausea) and two in arm B (one for nausea and one withdrew consent after the second visit because he didn't want to repeat all the required examinations) (Figure 2).

A total of 57 primary open angle glaucoma Caucasian subjects completed the study: 26 in group A and 31 in group B. The overall median age was 66.5 [61.3–73.2] years, 29 patients (51%) were female. Most patients had one or more systemic diseases, in particular 20 (36%) subjects had systemic hypertension, 15 (26.3%) hypercholesterolaemia, seven (12.3%) vascular disorders (i.e. acute myocardial or cerebral infarction, cardiomyopathy), four (7%) diabetes (Table 1). At baseline, most patients (44 subjects, 77%: 20 in group A and 24 in group B) had an early glaucomatous visual field defect, the others had a moderate defect; visual acuity was very good and intra-ocular pressure was generally well controlled (Table 2).

**Table 1:** Baseline demographic data of group A (current topical therapy + CIT/HOMO one tablet a day, in the first period) and group B (current topical therapy alone, in the first period).

	**Overall group** **(57 patients)**	**Group A** **(26 patients)**	**Group B** **(31 patients)**	***p*-value**
**Age (median [IQR] years)**	66.5 [61.3–73.2]	67.5 [63.2–72.1]	64.2 [60.7–68.8]	0.89
**Gender (Male/Female) (number, %)**	28 (49)	13 (50)	15 (48)	0.9
	29 (51)	13 (50)	16 (52)	
**Concomitant systemic diseases (N, %)**			
Systemic hypertension	20 (35.1)	10 (38.5)	10 (32.3)	0.62
Hypercholesterolaemia	15 (26.3)	6 (23.1)	9 (29)	0.61
Vascular disorders	7 (12.3)	4 (15.4)	3 (9.7)	0.51
Diabetes	4 (7)	1 (3.8)	3 (9.7)	0.39
Arteritis	3(5.3)	2 (7.7)	1 (3.2)	0.45
**Concomitant ocular refractive defects (N, %)**			
Emmetropia	13 (22.8)	5 (19.2)	8 (25.8)	0.18
Myopia	13 (22.8)	6 (23.1)	6 (19.3)	
Hyperopia	17 (29.8)	11 (42.3)	7 (22.6)	
Other	14 (24.6)	9 (34.6)	5 (16.1)	
**Topical therapy (N, %)**				
Fixed association	19 (33.3)	9 (34.6)	10 (32.3)	0.85
Monotherapy	38 (66.7)	17 (65.4)	21 (67.7)	0.76

**Table 2:** Baseline clinical characteristics of group A (current topical therapy + CIT/HOMO one tablet a day, in the first period) and group B (current topical therapy alone, in the first period).

	**Overall group** **(57 patients)**	**Group A** **(26 patients)**	**Group B** **(31 patients)**	***p*-value**
**Visual acuity (decimals, Snellen)**	9.5 ± 1.2	9.4 ± 1.1	9.5 ± 1.2	0.54
**IOP (mmHg)**	14.6 ± 2.5	14.2 ± 2.1	15.1 ± 2.7	0.07
**Visual field**				
VFI (%)	91.8 ± 8.3	90.7 ± 9	92.9 ± 7.7	0.21
MD (decibels)	−3.5 ± 3.3	−3.9 ± 3.6	−3 ± 3.1	0.16
PSD (dB)	4.1 ± 3	4.4 ± 3.2	3.9 ± 2.9	0.4
**Pattern electroretinogram (PERG) -transient PERG**				
**Central**				
Amplitude Amp-p50 (μV)	2.7± 2.1	2.5± 2.3	2.5± 1.9	0.29
Latency Lat-p50 (msec)	59.4± 8.2	60.2± 10.9	58.7± 4.7	0.34
Amplitude Amp-n95 (μV)	1.2± 2.5	1.6± 2.5	0.9± 2.4	0.17
Latency Lat-n95 (msec)	106.3± 22.9	107.8 ± 23.5	105± 22.4	0.53
**Inferior**				
Amplitude Amp-p50 (μV)	1.4 ± 1.3	1.1 ± 1.1	1.6 ± 1.3	0.07
Latency Lat-p50 (msec)	59.9 ± 8.4	60.5 ± 8.9	59.5 ± 8.2	0.61
Amplitude Amp-n95 (μV)	0.7 ± 1.1	1 ± 1.2	0.6 ± 0.9	0.08
Latency Lat-n95 (msec)	110.6 ± 10.5	111.6 ± 10.8	109.9 ± 10.4	0.51
**Superior**				
Amplitude Amp-p50 (μV)	1.1 ± 1.8	0.8 ± 2.3	1.3 ± 1.2	0.22
Latency Lat-p50 (msec)	60.3 ± 6.5	60.2 ± 5.8	60.3 ± 7.1	0.92
Amplitude Amp-n95 (μV)	1 ± 1.7	1 ± 2.3	1 ± 1.1	0.88
Latency Lat-n95 (msec)	111.1 ± 14.5	110.9 ± 16.4	111.1 ± 13	0.95
**PERG steady state (Hz)**				
**Central**				
Amplitude	1.8 ± 1.3	1.9 ± 1.4	1.7 ± 1.2	0.62
Latency	114 ± 29.5	108.2 ± 31.8	118.5 ± 27.4	0.14
**Inferior**				
Amplitude	1.2 ± 1	1.1 ± 0.9	1.3 ± 1.1	0.65
Latency	129.1 ± 9.7	130.5 ± 14.2	128.5 ± 7.1	0.56
**Superior**				
Amplitude	1.2 ± 1.1	1.6 ± 1.4	1 ± 1	0.17
Latency	130.5 ± 7.4	130.5 ± 8.5	130.5 ± 6.9	0.97
**Quality Of life (NEI-VFQ25)**				
General health	64 ± 15	65.9 ± 12.3	62.5 ± 16.9	0.39
General vision (GV)	68.1 ± 11	69.4 ± 11.8	67.1 ± 10.4	0.62
Ocular Pain–OP	79.4 ± 18.2	78.4 ± 19.9	80.2 ± 17	0.61
Near Activities–NA	79.5 ± 19.2	81.4 ± 19.8	77.9 ± 18.8	0.45
Distance Activities–DA	89.2 ±	89.7 ± 16.9	88.7 ± 9.5	0.51
Vision Specific Social Functioning–VSSF	94.3 ± 12.5	91.8 ± 15.8	96.4 ± 8.6	0.18
Vis Spec Mental Health–VSMH	77.8 ± 16.3	75.5 ± 15.2	79.8 ± 17.1	0.49
Vis Spec Role Difficulties–VSRD	91.4 ± 16.7	93.7 ± 16.3	89.5 ± 17.1	0.85
Vis Spec Dependency–VSD	94.6 ± 15.1	92 ± 19.6	96.8 ± 9.8	0.76
Driving–D	80.1 ± 23.6	82.8 ± 22	77.9 ± 24.9	0.54
Color vision–CV	95.2 ± 12	92.3 ± 15.4	97.6 ± 7.5	0.48
Peripheral Vision–PV	89 ± 18.3	90.4 ± 18.8	87.9 ± 18.1	0.56

*All values are expressed as mean ± SD*.

Baseline demographic and clinical characteristics were well balanced between the two arms (Tables 1, 2), with no statistically significant differences.

### Function

#### Electrophysiology

Regarding the transient PERG examination, at the end of the intake period, patients had P50 and N95-waves amplitude higher than those on topical therapy alone. Statistically significant increase was observed in the inferior and superior P50-waves amplitude, respectively of 0.47 μV (95% CI, 0.02–0.93; *p* = 0.04) and of 0.65 (95% CI, 0.16–1.13; *p* = 0.009), and in the inferior N95-wave amplitude of 0.63 μV (95% CI, 0.22–1.04; *p* = 0.002) (Table 3).

**Table 3:** Pattern electroretinogram (PERG), transient and steady state, examinations by period (pre and post): current topical therapy alone OR current topical therapy + CIT/HOMO one tablet a day.

	**Current topical therapy alone**		**Current topical therapy** **+** **CIT/HOMO**		**95% CI**	***p*-value**
	**Pre**	**Post**	**Pre**	**Post**		
**Pattern electroretinogram (P-ERG) transient**						
**Central**						
Amplitude Amp-p50 (μV)	2.6 ± 1.7	2.6 ± 1.9	2.6 ± 2	3.2 ± 7.4	0.7 (−0.75–2.12)	0.35
Latency Lat-p50 (msec)	59.1 ± 7.1	57.6 ± 7.5	59 ± 8.6	59.4 ± 7.7	2 (−0.31–4.32)	0.09
Amplitude Amp-n95 (μV)	0.9 ± 2.3	1.2 ± 2.3	1.1 ± 2.7	1.5 ± 1.8	0.2 (-0.68–0.71)	**0.05**
Latency Lat-n95 (msec)	105 ± 23.8	108.1 ± 22.6	105.5 ± 25.1	104.5 ± 22.8	−4.3 (−11.7–3)	0.25
**Inferior**						
Amplitude Amp-p50 (μV)	1.3 ± 1.3	0.9 ± 1.1	1 ± 1.1	1.2 ± 1.6	0.5 (0.02–0.93)	**0.04**
Latency Lat-p50 (msec)	57.5 ± 7.7	58.5 ± 1	59.1 ± 11	59.9 ± 8.3	−1 (−5.2–3.2)	0.66
Amplitude Amp-n95 (μV)	0.6 ± 0.8	1 ± 1.1	1.1 ± 1.2	1.1 ± 1.6	0.6 (0.2–1)	**0.002**
Latency Lat-n95 (msec)	111. ± 12.6	109.2 ± 15.2	108.3 ± 11.7	111.4 ± 12.8	4.1 (-1.9–10)	0.18
**Superior**						
Amplitude Amp-p50 (μV)	1.2 ± 1.3	0.9 ± 1.2	1 ± 1.7	1.3 ± 1.6	0.6 (0.2–1.1)	**0.009**
Latency Lat-p50 (msec)	59.2 ± 7.6	60.4 ± 6.3	60.7 ± 6.5	58.7 ± 7	−3.3 (-6–0.5)	**0.01**
Amplitude Amp-n95 (μV)	0.8 ± 1.1	1.1 ± 1.1	1.1 ± 1.7	1.2 ± 1.5	0.2 (−0.4–0.7)	0.55
Latency Lat-n95 (msec)	111.8 ± 14.1	108.5 ± 13	110.1 ± 15.7	113.2 ± 19	6.5 (−0.6–13.5)	**0.07**
**PERG Steady state (Hz)**						
**Central**						
Amplitude	1.5 ± 1.3	1.6 ± 1	1.8 ± 1.2	1.8 ± 1.4	−0.01 (-0.5–0.4)	0.96
Latency	115 ± 30	114.1 ± 29.2	113.6 ± 29.6	113.6 ± 29	0.9 (−1.8–3.5)	0.53
**Inferior**						
Amplitude	1.1 ± 1	0.7 ± 0.8	0.9 ± 0.9	1 ± 1.8	0.4 (-0.5–1.3)	0.34
Latency	129.3 ± 10.2	126.5 ± 12.6	127.6 ± 12	130.2 ± 10.3	5.3 (−2.6–13.2)	0.19
**Superior**						
Amplitude	0.9 ± 1.4	0.8 ± 0.9	1 ± 1.2	0.9 ± 1.6	−0.2 (−1.1–0.8)	0.74
Latency	129.3 ± 10.5	132 ± 11.9	132.3 ± 11.2	132.4 ± 10.1	−2.4 (−9.1–4.2)	0.47

*P-values refer to the comparison between treatments, significant values are reported in bold (see text for statistical analyses methods)*.

A significant shorter peak time of 3.3 μV (95% CI,−6.01–−0.54; *p* = 0.01) was observed only for superior P50 wave.

About Steady State PERG, no significant changes were recorded (Table 3).

All these effects were independent from age, gender, visual field defect, IOP values and kind of therapy.

#### Visual Field

Mean deviation was not statistically different between the periods in which patients took CIT/HOMO or not (Table 4); while PSD significantly improved (*p* = 0.003) when CIT/HOMO was added to topical therapy, independently from age, gender, kind of therapy.

**Table 4:** Visual acuity and visual field (VF) examination by period (pre and post): current topical therapy alone OR current topical therapy + CIT/HOMO one tablet a day.

	**Current topical therapy alone**		**Current topical therapy** **+** **CIT/HOMO**		**95% CI**	***p*-value**
	**Pre**	**Post**	**Pre**	**Post**		
**Visual acuity**	9.5 ± 1.2	9.5 ± 1.3	9.5 ± 1.2	9.5 ± 1.2	0.6 (−0.11–0.22)	0.51
**Visual field**						
VFI (%)	92.3 ± 8.8	91.3 ± 9.8	91.8 ± 8.8	91.4 ± 12.6	0.5 (−1.63–2.59)	0.66
MD (dB)	−3.4 ± 3.4	−3.8 ± 3.7	−3.7 ± 3.4	−3.7 ± 4.5	0.3 (−0.30–1.01)	0.29
PSD (dB)	4 ± 2.8	4.1 ± 2.9	4.2 ± 3.1	3.9 ± 3	−0.6 (−0.99–0.20)	**0.003**

*P-values refer to the comparison between treatments, significant values are reported in bold (see text for statistical analyses methods)*.

#### IOP

The mean IOP did not change significantly when patients took the study tablet of CIT/HOMO.

### Quality of Life

During the period in which CIT/HOMO mg was taken, patients had higher NEI-VFQ25 scores in all the subscales, reaching statistical significance only for the VSD item score (*p* = 0.04) (Table 5). Scores were independent from age, gender, visual field defect, IOP values and kind of therapy.

**Table 5:** Quality of Life examined by 25-item National Eye Institute-Visual Function Questionnaire by period (pre and post): current topical therapy alone OR current topical therapy + CIT/HOMO one tablet a day.

	**Current topical therapy alone**		**Current topical therapy** **+** **CIT/HOMO**		**95% CI**	***p*-value**
	**Pre**	**Post**	**Pre**	**Post**		
**NEI-VFQ 25**						
General Health–GH	64.1 ± 15.5	64.4 ± 13.6	64 ± 13.4	65.1 ± 13.1	0.8 (−2.6–4.2)	0.65
General Vision–GV	69.5 ± 10.6	69 ± 9.7	68.2 ± 10.3	68 ± 11.3	0.5 (−2.4–3.4)	0.73
Ocular Pain–OP	78.5 ± 18.7	77 ± 21.2	77.6 ± 20.8	78.5 ± 20.4	2.3 (−2.6–7.1)	0.36
Near Activities–NA	78.6 ± 19.2	77 ± 18.3	77.2 ± 19.1	77.3 ± 17.8	1.3 (−4–6.6)	0.63
Distance Activities–DA	89 ± 11.9	87.3 ± 12.4	87.6 ± 14.2	86.5 ± 13.8	0.6 (−2.6–3.9)	0.7
Vision Specific Social Functioning–VSSF	96.5 ± 9.4	96.3 ± 8.8	94.5 ± 12	92.8 ± 11.1	−1.2 (−4.6–2.1)	0.48
Vision Specific Mental Health–VSMH	77.7 ± 19.3	76 ± 18.7	76.9 ± 17.8	77.5 ± 15.8	2.3 (−2.5–7.2)	0.34
Vision Specific Role Difficulties–VSRD	90.8 ± 15.8	89.2 ± 19.5	91.9 ± 18.2	91.7 ± 15.5	1.40 (−3.5–6.3)	0.58
Vision Specific Dependency–VSD	94 ± 14.4	90.7 ± 20.2	91.8 ± 18.9	94.4 ± 13.9	5.8 (−0.0–11.6)	**0.04**
Driving–D	80.8 ± 23	83.7 ± 18	82.9 ± 19.1	81.1 ± 22.3	−4.3 (−9.5–0.9)	0.11
Color vision–CV	96 ± 10.3	95 ± 10.3	95.6 ± 11.7	93 ± 13.1	−2.3 (−6.5–1.8)	0.26
Peripheral Vision–PV	89 ± 17.7	87.7 ± 16.4	88.2 ± 17.1	86.8 ± 19.5	−0.1 (−4.6–4.5)	0.97

*P-values refer to the comparison between treatments, significant values are reported in bold (see text for statistical analyses methods)*.

### Adverse Events

Two (3.5%) patients reported mild nausea after taking the tablet of CIT/HOMO and asked to leave the study.

## Discussion

The present study found a positive significant effect of the oral fixed combination of Citicoline 500 mg plus Homotaurine 50 mg on the retinal function and the neural conduction along the visual system in stable primary open angle glaucoma patients with well controlled and stable intraocular pressure. Secondarily, our data pointed out an improvement in the visual field and in the quality of life, examined by NEI-VFQ 25, in the period of CIT/HOMO intake.

Glaucoma is a multifactorial and complex, degenerative and progressive disease of the optic nerve that affects not only the eye with a damage to the RGCs but also the postretinal visual pathways; therefore glaucoma is an eye disease that compromises also the integrity of the visual brain structures.

The increase in IOP is the main and best known risk factor for the development of glaucoma: several studies have shown and support that the reduction of IOP has a positive effect on the progression of the disease. Since glaucoma is a disease of the entire visual system, the reduction of IOP acts not only by reducing the topical compressive effect of pressure on the ocular structures but also as “indirect neuroprotection”: the IOP reduction limits the neurodegenerative processes of the retinal ganglion cells and, backwards, of the cells of the visual system.

A recent review by Gandolfi (21) resumes the pathogenesis of the visual defect in glaucoma. Briefly, the elevated intraocular pressure causes strain and stress on the axons of the RGCs disrupting the axonal transport. The damaged RGCs cause a process of trans-synaptic degeneration, independent from IOP values, that affects the extracellular microenvironment and the other retinal cells, involving the mitochondrial pathway and leading to programmed cell death, called apoptosis, and optic neuropathy.

Previous literature supports the fact that the degeneration of the RGCs is due to several events, some of which have been known for years such as increased IOP, oxidative stress, glutamate excitotoxicity, ischemia or impaired microcirculation; while others have recently been recognized: mitochondrial dysfunction, autoimmune dysregulation, activation of neurotrophic growth factor, activation of microglia and astrocytes, protein misfolding (16, 22, 23).

All these evidence support the need for novel therapeutic approaches to act early on glaucoma progression using drugs other than IOP-lowering molecules with the aim of improving and modulating neuronal and retinal cell survival.

Some new substances have been studied for the treatment of glaucoma and others are being studied: to date, citicoline has been widely recognized as having neuroprotective properties in glaucoma. It is a precursor of the neurotransmitter acetylcholine and other components of the neuronal membrane such as phosphatidylcholine and sphingomyelin. Citicoline (cytidine-5′-diphosphocholine) mediates neurodegenerative events by restoring membrane integrity (24), improving mitochondrial function (25), improving axonal transport deficits (13), reducing glutamate excitotoxicity (26) and oxidative stress (27), elevating the neurotrophin level (13).

There is therefore evidence that citicoline acts as a neuroprotector, but it is now equally evident that the mechanisms underlying ganglion cell apoptosis are multiple and therefore it is assumed that the neuroprotection of ganglion cells may also require the use of different molecules able of act synergistically on the causes of apoptosis.

In the present study, a fixed combination of citicoline and homotaurine was investigated.

Homotaurine (3-aminopropanesulfonate) is a small natural aminosulfonate compound identified in various species of red marine algae and subsequently chemically synthesized and introduced into clinical use under the name of tramiprosate. It has been shown to have a wide range of effects (14). Experimental studies, *in vitro* and in vivo, have shown that homotaurine can interfere with various cellular pathways due to the activation of GABA type A receptors: it exerts effects against oxidative damage to DNA, antifibrillogen activity, antinociceptive and analgesic activity, finally acting as a neuprotector and neutrotropic molecule (14, 28). It also reduces amyloid aggregation by preventing the neurotoxicity of the Aβ peptide (29).

The literature has highlighted a potential role of amyloid in the development of RGC apoptosis in experimental models of glaucoma (30, 31) making homotaurine potentially useful as a potentially effective treatment in glaucoma.

A recent study showed that the fixed combination of citicoline and homotaurine exhibits potent neuroprotective activities through distinct molecular mechanisms, demonstrating a synergistic effect, in cultured retinal cells, of the two compounds in reducing the proapoptotic effects associated with exposure to both glutamate and high doses of glucose (17).

The present study examined the effects of this fixed combination *in vivo* to evaluate / test its usefulness also in clinical practice.

We hypothesized that PERG could represent the most indicative test to verify a potential effect of the fixed combination of citicoline and homotaurine on the function of internal retinal cells, given that for years it has been known that glaucoma causes a dysfunction of the inner retinal layers with consequent abnormal responses in the PERG recordings (32–34), that can be reversible (35).

Therefore, the main purpose of the study was precisely to investigate whether there was, *in vivo*, a positive effect of the assumption of this new association on internal retinal function, recording amplitudes and latencies of the main waves of the PERG recordings.

The literature data on the neuroprotective effects related to the intake of citicoline alone on the PERG refer to the research of Parisi and collaborators who tested the effectiveness of the intake of citicoline 1,000 mg by intramuscular injection (9) and the effects of the intake of 1,600 mg of citicoline orally (36). Authors recorded improvement both in the latency of p50 wave and in the N95 amplitude (Table 6). Our data are in agreement with these observation; in addition, a significant improvement in the amplitude of the p50 wave was also recorded in our patients, indicating an improvement in ganglion cell function.

**Table 6:** Comparison of our data and data from literature.

	**Our data** **(57 patients)**		**Parisi (7)** **1,000 intramuscular** **(25 patients)**		**Parisi (36)** **1,600 oral route** **(20 patients)**	
	**Pre**	**Post** **(4 months)**	**Pre**	**Post** **(60 days)**	**Pre**	**Post** **(2 months)**
**Pattern Electroretinogram (P-ERG) transient**						
**Central**						
Amplitude Amp-p50 (μV)	2.6 ± 2	3.2 ± 7.4	NA	NA	NA	NA
Latency Lat-p50 (msec)	59 ± 8.6	59.4 ± 7.7	69.3 ± 2.8	62.3 ± 3.1	70 ± NA	62.5 ± NA
Amplitude Amp-n95 (μV)	1.1 ± 2.7	1.5 ± 1.8	0.7 ± 0.3	1 ± 0.4	1 ± NA	1.5 ± NA
Latency Lat-n95 (msec)	105.5 ± 25.1	104.5 ± 22.8	NA	NA	NA	NA
Visual Field
PSD (dB)	4.2 ± 3.1	3.9 ± 3	NA	NA	NA	NA

By directly comparing our data with those reported by Parisi on the amplitude of N95 and the latency of P50, we observed one thing worth noting: the extent of the improvement obtained was similar to that observed by the other researchers but this effect was obtained by us with less than half a dose of citicoline (500 mg). In our opinion, having obtained numerically comparable improvement to that of our colleagues using a reduced dose of citicoline could be an indirect evidence of the synergistic effect of homotaurine added to citicoline, confirming the usefulness of using different molecules with different mechanisms of action in the prevention of apoptosis and in the development of neuromodulation.

Obviously these data must be confirmed, with studies conducted on a greater number of patients and perhaps with studies that use the individual components, although we are very encouraged to observe that the association of 500 mg of citicoline with 50 mg of homotaurine allows to obtain the same results that are obtained with 1,000 mg of citicoline in the muscle or 1,600 mg orally, with an economic saving for the patient who has to take a lower dose of citicoline to obtain the same result.

It should be noted that our study differs from the previous ones by Parisi et al. on electrophysiology for 2 characteristics: 1. we examined both the transient and the stationary (steady state, SS) PERG component and 2. the instrument we used, the Retimax, allowed us to examine not a single response of the PERG but the response for 3 quadrants (upper, lower and central).

Out data showed that the more peripheral (lower and upper) contingents of RGCs improve in transient PERG registration during the period in which patients take NP, both in the luminance component (P50) and in the contrast component (N95). This improvement concerns an improvement of the areas that are typically altered in glaucoma which is a pathology that mainly affects the periphery and not the center (upper and lower fibers, with typical upper and lower arciform perimetic defects).

The steady state gives a stable response: before this is altered, the center must also have greater impairment, our patients did not have a central impairment as shown by the inclusion/exclusion criteria, this could explain the discrepancy between PERG transient improvement and stability of the SS. We suppose that, for the central ones to improve, longer observation periods are likely required. The stable component (SS) is less influenced then the transient one, by definition. In fact, the response recorded from the steady state is a frequency response, it is a stable response.

However, regarding electrophysiological changes recorded, what can be stated with certainty is that the neuroprotective effect on the PERG trace is due to the assumption of the fixed association under study and is independent of a possible effect linked to indirect neuroprotection due to IOP reduction, given that the IOP did not undergo significant changes when taking citicoline and omotaurine.

Regarding our secondary endpoints, MD remained stable and PSD significantly improved during oral intake of CIT/HOMO: these observations support the potential neuroprotective effect of these molecules, as it is known that PSD may be a useful index for detecting the progression of glaucoma that is equal to, or even better than, MD (37).

Our data are in agreement with previous literature which found a reduction in the mean progression rate of MD after 2 years of treatment with Citicoline (8, 36), although our patients had better visual field defects (mean MD value at baseline: −3.5 ± 3.3 dB) compared to the sample reported in the literature (mean MD value at baseline: −9.2 ± 6.7 dB) and therefore the results are not fully comparable. It is in fact widely known that the sensitivity values of the visual field depend on the subjective visual detection of the stimuli, and therefore are influenced by various factors in addition to the RGCs such as irregularities of the pre-retinal media, integrity of the visual system, greater visual processing, time of reaction and attention, and the severity of the visual field defect affects all of these factors. Some Authors have described a possible increase in the consciousness level (38) of subjects during the assumption of citicoline and this could explain the improved psychophysical responses evaluated by visual field analysis observed in our patients, even if, using electrophysiological methods, it was observed that citicoline may induce an enhancement of the ganglion cell function and neural conduction along the postretinal visual pathways leading to an improvement of visual function in glaucoma independently from the increase in the consciousness level (36).

A final comment in this regard is inspired by a recent work on the CIT/HOMO association: the study showed an improvement in contrast sensitivity in the period in which glaucoma patients took the CIT/HOMO association. Although the precise mechanism of action is unclear, it has been hypothesized that the effect is likely due to both its dopaminergic action and a direct action of the retina (39). An improvement in contrast sensitivity could, in part, explain an improvement in the execution of the visual field and therefore in its result.

However, it should be kept in mind that any variation on visual field changes requires several years of observations to establish a reliable trend as reported in the work of Krupin et al. (40) therefore the observed data on PSD variation are interesting but need to be further investigated. Infact, even if our study noted some effects on the visual field, the possible effect on the visual field was a secondary, exploratory aim, and the study was not powered to detect this.

Finally, regarding the vision-related quality of life, its role is increasingly central in the management of glaucoma, so much so that the concept of quality of life is part of the definition of the therapeutic objectives of glaucoma in the guidelines drawn up by the European Glaucoma Society who recognized that the goal of treatment is the preservation of visual function and quality of life. The methods of improving this are not yet fully understood, and the challenge of the researchers is to identify molecules that can improve patients' vision related quality of life. A previous paper has shown that the fixed association CIT/HOMO can improve the quality of life as examined with the GQL-15 questionnaire (39), our data are in agreement with these observations even if the questionnaire used to evaluate quality of life was different.

In general we found that all the scales improved during the intake of citicoline and omotaurine, even if only one significantly: after all, it is known that the quality of life in glaucoma is strongly influenced by the severity of the perimetric defect and having 80% of our patients with an initial perimeter defect, we did not expect to see large effects on quality of life.

In fact, the only scale that has significantly improved is that of “vision specific dependency”, this in our opinion is very important because the intake of these molecules has been able to reduce the symptoms of dependence of patients on other people and therefore to give patients a greater sense of independence from others since it has improved symptoms related to these three statements: 1. “I stay at home most of the time because of my vision”; 2. “due to my vision, I have to depend too much on what other people tell me”; 3. “I need a lot of help from others because of my vision”.

Of note that the impact of vision-related quality of life on glaucoma patients is widely recognized: our study suggests that vision-related quality of life may change, improving, while taking one tablet a day of CIT/HOMO, even if the absence of a placebo group could be a limit in the quality of life assessment. A possible pathogenetic hypothesis could lie in an effect of citicoline and homotaurine at the CNS level that could improve patients' perception of quality of life but there are still no specific studies on these two molecules.

Anyway, a specific study aimed and powered at assessing the quality of life should be done to confirm our data, considering a longer follow up period.

Our study has limits, some have already been listed, we want to remember here the two main limits which are: the first is that we examined a fixed combination and therefore we are not able to quantify the effects of the individual components; the second is that the study has a short follow-up and a longer study is mandatory to evaluate effects on long term of these potential neuroenhancer and neuromodulators since glaucoma is a chronic disease and the therapy should be chronical too.

## Conclusion

Daily oral intake of the fixed combination of citicoline 500 mg and homotaurine 50 mg for 4 months improved the function of inner retinal cells recorded by PERG independently from IOP reduction and positively influenced the visual field and the perception of quality of life.

This interesting observation needs to be verified but represents a valid option for practicing neuromodulation in patients with glaucoma to prevent disease progression.

## Data Availability Statement

The raw data supporting the conclusions of this article will be made available by the authors, without undue reservation.

## Ethics Statement

The studies involving human participants were reviewed and approved by Ethics Committee of the IRCCS Policlinico San Matteo Foundation of Pavia (prot. 2015000565). The patients/participants provided their written informed consent to participate in this study.

## Author Contributions

GR wrote the main manuscript text. AP and BS did PERG and did visual fields. GR analyzed PERG test. AD performed the statistical analysis. GR, AVM, TR, DS, and AM collected the data. EP randomized patients. GP and GM entered the data into the database. GP prepared tables and figures. All authors reviewed the manuscript.

## Conflict of Interest

The authors declare that the research was conducted in the absence of any commercial or financial relationships that could be construed as a potential conflict of interest.

## Publisher's Note

All claims expressed in this article are solely those of the authors and do not necessarily represent those of their affiliated organizations, or those of the publisher, the editors and the reviewers. Any product that may be evaluated in this article, or claim that may be made by its manufacturer, is not guaranteed or endorsed by the publisher.
